# Benchmarking of two bioinformatic workflows for the analysis of whole-genome sequenced *Staphylococcus aureus* collected from patients with suspected sepsis

**DOI:** 10.1186/s12879-022-07977-0

**Published:** 2023-01-20

**Authors:** Mahnaz Irani Shemirani, Diana Tilevik, Andreas Tilevik, Sanja Jurcevic, Dimitrios Arnellos, Helena Enroth, Anna-Karin Pernestig

**Affiliations:** 1 School of Bioscience, Systems Biology Research Centre, Infection Biology, University of Skövde, Skövde, Sweden; 2 1928 Diagnostics, Gothenburg, Sweden; 3 Molecular Microbiology, Laboratory Medicine, Unilabs AB, Skövde, Sweden; 4 Department of Laboratory Medicine, Institute of Biomedicine, Sahlgrenska Academy, University of Gothenburg, Gothenburg, Sweden

**Keywords:** Whole-genome sequencing, Antimicrobial susceptibility, *S. aureus*, Species identification, Virulence genes, Clinical microbiology, Illumina sequencing, Benchmarking

## Abstract

**Background:**

The rapidly growing area of sequencing technologies, and more specifically bacterial whole-genome sequencing, could offer applications in clinical microbiology, including species identification of bacteria, prediction of genetic antibiotic susceptibility and virulence genes simultaneously. To accomplish the aforementioned points, the commercial cloud-based platform, 1928 platform (1928 Diagnostics, Gothenburg, Sweden) was benchmarked against an in-house developed bioinformatic pipeline as well as to reference methods in the clinical laboratory.

**Methods:**

Whole-genome sequencing data retrieved from 264 *Staphylococcus aureus* isolates using the Illumina HiSeq X next-generation sequencing technology was used. The *S. aureus* isolates were collected during a prospective observational study of community-onset severe sepsis and septic shock in adults at Skaraborg Hospital, in the western region of Sweden. The collected isolates were characterized according to accredited laboratory methods i.e., species identification by MALDI-TOF MS analysis and phenotypic antibiotic susceptibility testing (AST) by following the EUCAST guidelines. Concordance between laboratory methods and bioinformatic tools, as well as concordance between the bioinformatic tools was assessed by calculating the percent of agreement.

**Results:**

There was an overall high agreement between predicted genotypic AST and phenotypic AST results, 98.0% (989/1006, 95% CI 97.3–99.0). Nevertheless, the 1928 platform delivered predicted genotypic AST results with lower very major error rates but somewhat higher major error rates compared to the in-house pipeline. There were differences in processing times i.e., minutes versus hours, where the 1928 platform delivered the results faster. Furthermore, the bioinformatic workflows showed overall 99.4% (1267/1275, 95% CI 98.7–99.7) agreement in genetic prediction of the virulence gene characteristics and overall 97.9% (231/236, 95% CI 95.0–99.2%) agreement in predicting the sequence types (ST) of the *S. aureus* isolates.

**Conclusions:**

Altogether, the benchmarking disclosed that both bioinformatic workflows are able to deliver results with high accuracy aiding diagnostics of severe infections caused by *S. aureus*. It also illustrates the need of international agreement on quality control and metrics to facilitate standardization of analytical approaches for whole-genome sequencing based predictions.

**Supplementary Information:**

The online version contains supplementary material available at 10.1186/s12879-022-07977-0.

## Background

Infectious diseases caused by bacteria are one of the leading causes of human mortality and morbidity throughout the world, being responsible for several million deaths each year [1]. One of the most common pathways to death following an infection is sepsis, which arises when the body’s systemic response to an infection injures its own tissues and organs. It can lead to multiple organ dysfunction, shock and death, especially if not recognized early and treated promptly. Every hour of delayed appropriate antibiotic therapy increases mortality in septic shock by 5–10% [2, 3]. Early identification of patients having bacterial sepsis along with timely determination of causative bacteria and antibiotic resistance profiles can alter current practices for therapeutic management, reduce over-prescription of antibiotics and associated adverse outcomes [4, 5]. The rapidly increasing area of next-generation sequencing (NGS) technologies and more specifically bacterial whole-genome sequencing (WGS) could offer several applications in clinical microbiology including accurate and earlier species identification of bacteria, prediction of antimicrobial resistance and virulence genes [6]. However, bacterial WGS has seen slow integration into routine microbiological diagnostics because of the lack of a platform that can translate WGS data into clinical practice. Furthermore, WGS workflows are required to be standardized when considering the clinical diagnostic application [7]. To comply with the aforementioned points the commercial cloud-based platform, 1928 platform (1928 Diagnostics, Gothenburg, Sweden) was benchmarked against an in-house developed bioinformatic pipeline (INH) as well as to reference methods in the clinical laboratory. In the present study WGS data were retrieved from 264 *Staphylococcus aureus* isolates, using the Illumina HiSeq X next-generation sequencing technology. The *S. aureus* isolates were collected and characterized according to accredited laboratory methods during a prospective observational study of community onset of severe sepsis and septic shock in adults at Skaraborg Hospital, in the western region of Sweden [8]. The outcomes from the clinical laboratory methods, i.e., species identification by MALDI-TOF MS (Bruker) analysis and phenotypic antibiotic susceptibility testing (AST) following the EUCAST guidelines, were viewed as reference results, the true results, for the comparison with the genome-based computationally predicted output from the bioinformatic analyses of the WGS data. In addition, the virulence gene predictions obtained from the 1928 platform were compared to those obtained from the INH. Results for multi-locus sequence typing (MLST) were also investigated. Such application of WGS bioinformatics methods aiding in *S. aureus* diagnostics has also been addressed in other studies [9–11]. A recent study, analyzing WGS data from blood culture isolates of *S. aureus* using Next Gen Diagnostic software (Mountain View, California, USA) and the 1928 platform reported slightly high very major error (VME) and major error (ME) rates for the 1928 platform [12]. One VME is defined as a resistant phenotype with genetic predicted susceptible genotype, also known as false negatives. One ME is defined as a susceptible phenotype with genetic predicted resistant genotype, also known as false positives [13]. Since false negatives can have consequences for treatment of infection [14, 15] the present study compared the numbers of VME and ME, but also the VME and ME rates retrieved from the investigated bioinformatic workflows. Furthermore, the 1928 platform has been used in two studies focused on *S. argenteus* [16, 17] and in another study investigating the regional epidemiology and susceptibility patterns of methicillin resistant *S. aureus* (MRSA) isolates identified in Stockholm County, Sweden [18]. Lastly, a recent publication looking into the biodiversity of clinical *Klebsiella* spp. isolates collected from patients with suspected community-onset sepsis, Sweden, included bioinformatic analyses by the 1928 platform [19]. The present study aimed at to further evaluate the performance of the 1928 platform in clinical routine for in silico species identification, antibiotic susceptibility testing, virulence and sequence typing of *S. aureus.* Since rapid extraction of clinically relevant genomic information will be essential for the adoption of WGS for infection control and public health, the processing time of the bioinformatic workflows was also compared.

## Methods

### Bacterial isolates

**Fig.1:**
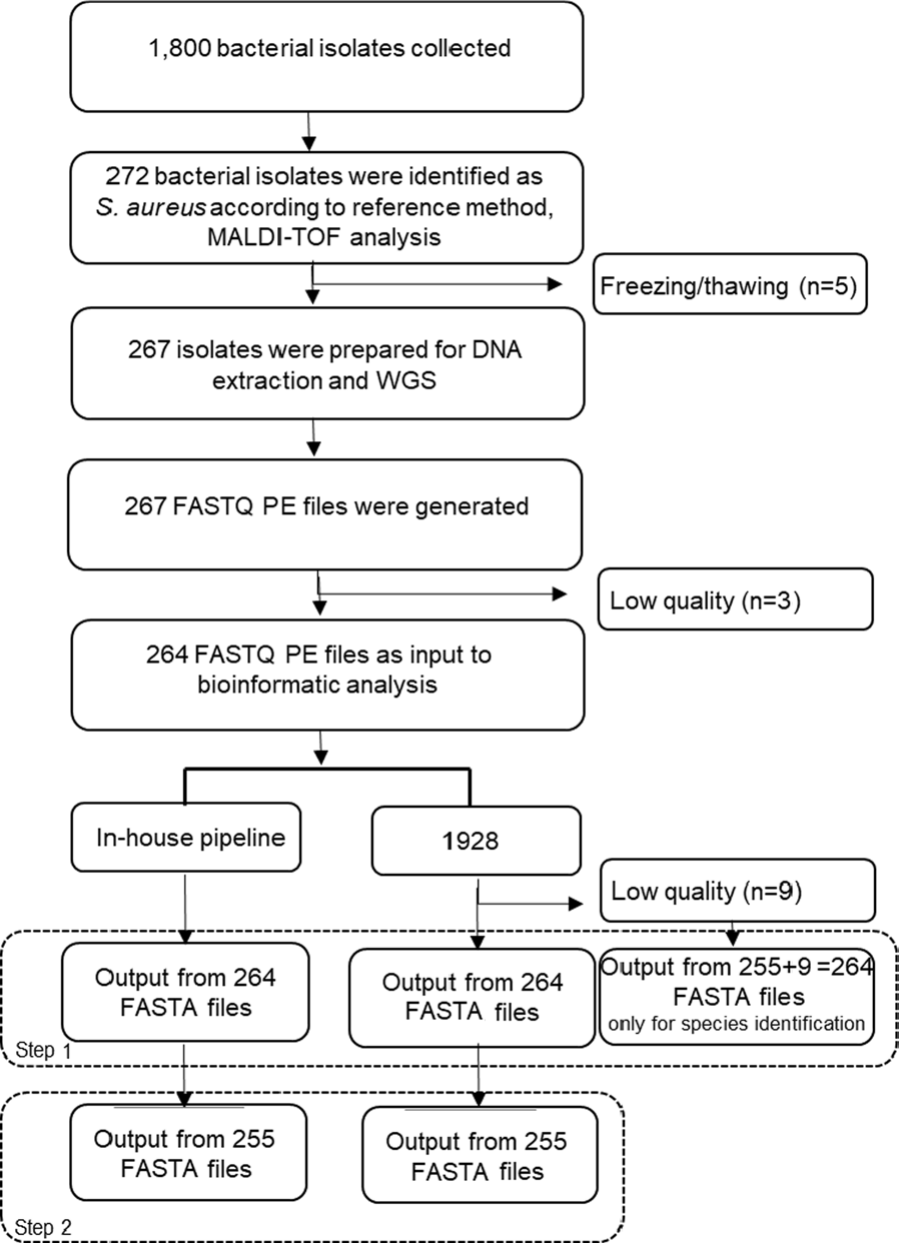
Overview of the bacterial isolates in the study. During a prospective observational study of community-onset severe sepsis and septic shock in adults conducted at Skaraborg Hospital, Sweden Ljungstrom [8] approximately 1,800 bacterial isolates were recovered. Definite species identification of the collected isolates was performed by MALDI-TOF MS, identifying 272 bacterial isolates as *S. aureus.* Five isolates could not be recovered after freezing. In all, 267 isolates were prepared for DNA extraction and WGS. The output FASTQ pair-ended (PE) files for three of the isolates were excluded from the dataset after quality control of the raw data and the remaining 264 *S. aureus* were used as input into the bioinformatic analysis in the in-house pipeline and 1928 platform. During the benchmarking of species identification (step 1) nine FASTQ files did not pass the quality control levels in the 1928 platform. Only when benchmarking species identification, the depth/coverage was lowered to 11-29X. During the benchmarking of antibiotic sensitivity, virulence genes and ST (step 2) output from 255 isolates identified as *S. aureus* both phenotypically and genotypically were included

From September 2011 to June 2012, a prospective observational study of community-onset severe sepsis and septic shock in adults was conducted at Skaraborg Hospital, a secondary hospital with 640 beds, in the western region of Sweden [8]. The study was approved by the Regional Ethical Review Board of Gothenburg (376–11). As the present study only focused on bacterial isolates recovered from cultures included in the routine patient care, individual informed consent is deemed unnecessary according to national regulations (2003:460). Approximately 1,800 bacterial isolates were recovered at the clinical microbiology laboratory, Unilabs, Sweden, from the patients enrolled in the study. These isolates were cryopreserved at the time of recovery by transferring colonial material to Microbank™ vials (Pro-Lab Diagnostics, Ontario, Canada) stored at − 80 °C. For the present study, isolates recovered from 212 patients and identified as *S. aureus* (n = 272) with routine microbiological methods based on cultures followed by MALDI-TOF MS (DB-4110) were selected. Nevertheless, five isolates could not be recovered after freezing. In all, 267 isolates were prepared for DNA extraction and WGS (Fig. 1).

### Reference method for species identification

In line with the hospital’s policy, blood cultures were drawn from each patient before the initiation of intravenous antibiotic treatment. For patients with sepsis of unknown origin, samples from urine and respiratory tract were cultured whenever possible. Other samples were collected at the discretion of the treating physician. Microbiological culturing was performed as previously described [20]. Definite species identification of the collected isolates was performed by MALDI-TOF MS on a Microflex LT mass spectrometer (Bruker Daltonics, Leipzig, Germany) using BioTyper software v2.0 using default parameter settings as described elsewhere [20, 21]. Spectral scores above 2.0 were used as a cut-off for correct identification. At the time of the study, the Bruker microorganism database MBT Compass Library DB-4110 (Bruker Daltonics, Germany) released in April 2011 was used.

### Reference method for antibiotic susceptibility testing

Antibiotic susceptibility was determined by accredited laboratory methods using the disc diffusion method on Mueller Hinton media according to European Committee on Antimicrobial Susceptibility Testing (EUCAST) guidelines (www.eucast.org). Antibiotic susceptibility test (AST) results retrieved from the identified *S. aureus* were included, hereafter referred to as phenotypic AST. Resistant AST result for isoxazolyl penicillin and cefoxitin was followed by detection of *mecA* by PCR to confirm the isolate as a methicillin resistant *S. aureus* (MRSA). Phenotypic AST results reported in this study are limited to the set of antibiotics included in the 1928 platform (Table 1).

**Table 1:** Genes and antibiotics assessed for each analysis in the 1928 platform accessed online June-July 2019

Antibiotics addressed for predicted genotypic antibiotic susceptibility^a^	Genes present in any of the SCCmec types		Typing-genes used for MLST classification	Virulence genes
Ciprofloxacin	*IS1272*	*ccrB3*	*arcC*	*etA*
Vancomycin	*ccrA1*	*ccrB4*	*aroE*	*etB*
Clindamycin	*ccrA2*	*ccrB6*	*glpF*	*lukF-PVL*
Erythromycin	*ccrA3*	*ccrC*	*gmk*	*lukS-PVL*
Isoxazolyl penicillin^b^	*ccrA4*	*mecA*	*pta*	*tsst1*
Rifampicin	*ccrB1*	*mecC*	*tpi*	
Trimethoprim	*ccrB2*		*yqiL*	
Tetracycline				
Fusidic acid				

^a^Phenotypic AST results reported in this study are limited to the set of antibiotics included in the 1928 platform

^b^Isoxazolyl penicillin belongs to the β-lactam antibiotic group

### Whole-genome sequencing of *S. aureus*—Illumina HiSeq

Genomic DNA was extracted at Unilabs, Skövde, using the MagNA Pure 96 DNA and Viral NA Small Volume kit (Roche Diagnostics, Switzerland) with the Pathogen Universal 200 protocol on a MagNA Pure 96 instrument (Roche Diagnostics, Switzerland). DNA concentration was measured using the Qubit dsDNA HS assay kit (Thermo Fisher Scientific, USA) on a Qubit 3.0 (Thermo Fisher Scientific, USA) and NanoDrop spectrophotometer, respectively (Thermo Fisher Scientific, USA). DNA extracts from 267 *S. aureus* isolates were transported to Clinical Genomics, SciLifeLab, Solna, Sweden, where the WGS was performed. During the sample preparation Nextera XT DNA sample preparation guide (Illumina, USA), was followed. Measurement of double-stranded DNA concentration was achieved with broad- and low range assay kits on a Qubit 2.0 (Thermo Fisher Scientific, USA). Library preparation was performed according to the Nextera XT guidelines (Illumina, USA). Fragment analyses of the PCR libraries on a Bioanalyzer (Agilent technologies, USA) was done to obtain abundances and average length of fragments for each sample. The Illumina HiSeq 2500 platform was used for the NGS. The output files consisted of compressed FASTQ-files (.gz) containing sequencing data that could be downloaded for further analysis.

### Bioinformatic analysis

**Fig. 2:**
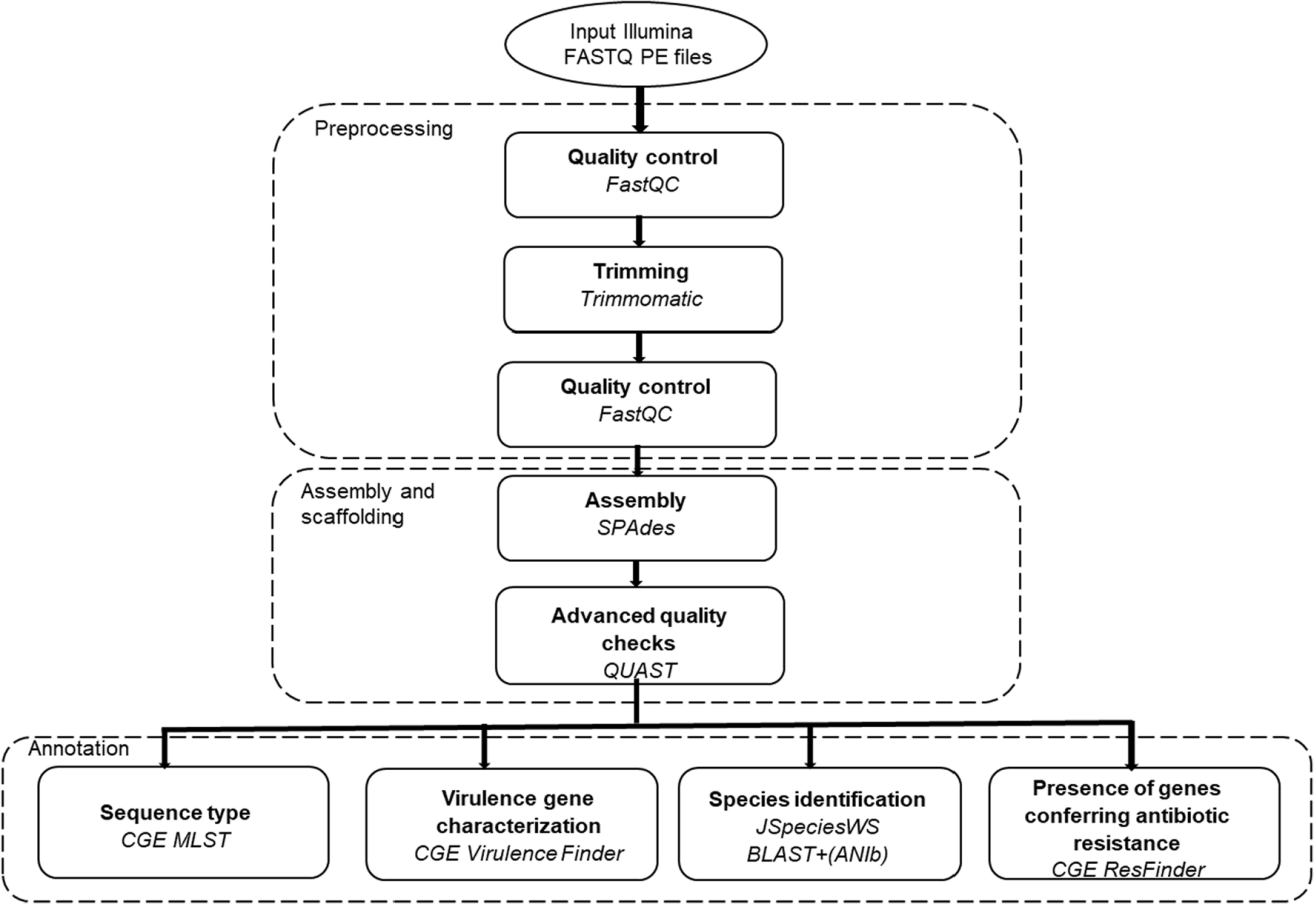
Overview of the in-house pipeline. The in-house pipeline consists of a number of manual steps; input of raw data, preprocessing of paired-end (PE) FASTQ files, assembly and scaffolding followed by annotation of the assembled contigs in FASTA format. The outputs from the annotation; sequence type (MLST), virulence gene characterization (VirulenceFinder), species identification (JSpeciesWS) and presence of genes conferring antibiotic resistance (ResFinder) were manually sorted and collected into a summary (Excel-format). Circle represents data files and each box represents a component corresponding to a series of tasks that provide a certain well-defined functionality (indicated in bold). Bioinformatics tool employed in each module are also mentioned (indicated in italics)

The INH consisted of established bioinformatic tools as illustrated in Fig. 2. In more detail, primary quality control of the FASTQ files was performed using the FastQC software (v.0.11.8) [22]. One isolate was removed from the dataset before trimming as it showed low number of reads (< 500,000). Trimmomatic (v.0.36) was used for adapter removal and quality trimming with a sliding window of size 4 and a minimum quality of 20 [23]. In addition, the first 12 bases were trimmed by the HEADCROP argument, and reads with a length shorter than 30 bp were removed. FASTQ files were then assembled into contigs using SPAdes (v.3.13.1) [24]. The quality of the assembled contigs were evaluated using the QUAST (v.5.0.2) [22] with default settings. In addition to determining assembly metrics, the length of each assembly was manually compared to the genome size of a reference genome obtained from NCBI. The reference genome used was *Staphylococcus aureus* subsp. *aureus*, NCTC 8325 (GenBank accession number NC_007795.1) with a genome size of 2.8 Mbp. If an assembly was not considered good, the median coverage was also calculated using R v.3.5 [23]. Two genome assemblies had a median coverage < 2.5 reads per base and were excluded from further analysis. The assembled contigs in FASTA format were annotated by tools available in the Center for Genomic Epidemiology (CGE) (https://www.genomicepidemiology.org/) i.e., ResFinder v.3.0 [25], VirulenceFinder v2.0 [26], MLST 2.0 [27] and the JSpeciesWS (http://jspecies.ribohost.com/jspeciesws/) (Fig. 2). Species identification was achieved by calculating the pairwise average nucleotide identity (ANI) based on BLAST + (ANIb) in JSpeciesWS [28] using *Staphylococcus aureus* subsp. *aureus* NCTC 8325 as the reference genome. An ANI threshold of 96% or greater was considered to delineate species boundaries as a threshold of 96% correlates well to DNA-DNA hybridization [28, 29] (Fig. 2). The presence of antibiotic resistance genes was predicted using CGE ResFinder v.3.0 with default settings for threshold ID (90%) and minimum length 60% [25]. Susceptibility was conferred by the absence of resistance genes and resistance was conferred by the presence of resistance genes. Presence (P) or absence (A) of virulence genes were predicted by CGE VirulenceFinder v2.0 [26] with default settings for threshold ID (90%) and minimum length 60%. MLST analysis was performed using CGE MLST 2.0 [27] with *Staphylococcus aureus* as selected configuration for all isolates. This MLST scheme consists of alleles from the following seven loci *arcC*, *aroE*, *glp*, *gmk*, *pta*, *tpi*, and *yqiL* [30]. For analysis with 1928, the FASTQ files were uploaded to its cloud-based platform (1928 Diagnostics, Sweden) for inferred antibiotic susceptibility based on genotype resistance markers, which are genes and mutations known to contribute to antibiotic resistance, hereby and later referred to as predicted genotypic antibiotic susceptibility. Susceptibility was conferred by the absence of genotype resistance markers and resistance was conferred by the presence of genotype resistance markers. The platform also predicts acquired virulence genes, type of mobile genetic SCCmec element and sequence type (Table 1), hereby and later referred to as predicted genotypic presence (P) or absence (A) of different virulence genes and ST, respectively. After uploading the FASTQ files, the files underwent an initial quality control were reads were trimmed or entirely discarded according to the platform’s internal thresholds i.e., sequencing depth/coverage higher than 30 × to perform the analysis. During species identification, analysis depth/coverage of 11-29X was allowed. Species identification, gene and mutation detection for the other analyses were performed by assembly free kmer-based methods (Table 2). Raw pair-end fastq.gz files were uploaded to the 1928 platform during June and July 2019. This platform has not been further updated during the access period as confirmed by communication with 1928 Diagnostics, Sweden.

**Table 2:** Analyses used during the benchmarking

Analyses compared during the benchmarking	Reference method Phenotypic results	Bioinformatic workflows Genotypic predicted results	
		1928	INH
Species identification	MALDI-TOF MS	kmer-based method^b^	JSpeciesWS
Antibiotic susceptibility test	Disc diffusion	kmer-based method^b^	ResFinder
Virulence genes	nd^a^	kmer-based method^b^	VirulenceFinder
Sequence type	nd^a^	kmer-based method^b^	MLST

^a^not determined, ^b^the underling method used is proprietary, the exact method cannot be mentioned, INH: in-house pipeline

### Benchmarking of the bioinformatic workflows

Species identification and AST results retrieved from the reference method in the clinical laboratory were compared to genetic predicted species identification and AST by both bioinformatic workflows through examining the degree of agreement between these results (Table 2). Nine antibiotics and/or antibiotic classes were included in the benchmarking (Table 3). A very major error (VME) was defined as a resistant phenotype with genetic predicted susceptible genotype, a major error (ME) was defined as a susceptible phenotype with genetic predicted resistant genotype [13]. Acquired virulence genes, presence (P) or absence (A), and STs retrieved from the bioinformatic workflows were compared by examining the degree of agreement between the genetically predicted results.

**Table 3:** Antibiotic groups included in the benchmarking

Disc diffusion Phenotypic AST^a^	Bioinformatic workflows Genotypic predicted AST	
	1928	INH
Ciprofloxacin	Ciprofloxacin	Ciprofloxacin
Clindamycin	Clindamycin	Lincosamide
Erythromycin	Erythromycin	Macrolide
Rifampicin	Rifampicin	Rifampicin
Trimethoprim	Trimethoprim	Trimethoprim
Tetracycline	Tetracycline	Tetracycline
Vancomycin	Vancomycin	Glycopeptide
Fusidic acid	Fusidic acid	Fusidic acid
Isoxazolyl penicillin	Isoxazolyl penicillin	Isoxazolyl penicillin

^a^Phenotypic AST results reported in this study are limited from the set of antibiotics included in the 1928 platform. As the phenotypic AST was performed as part of the routine clinical practice, the sample type mainly determined which antibiotics to be tested for each bacterial isolate (Additional file 1)

### Statistical analysis

Statistical analyses and calculations were performed using R v.4.0.3 [31]. Concordance between conventional microbiological methods and bioinformatic analysis tools was assessed by calculating the percent of agreement. Concordance between bioinformatic analysis tools was assessed by calculating the percent of agreement. The Agresti-Coull method was used [32] for the construction of 95% CI for percent of agreement between methods. Jupyter notebook v.6.0.3 [33] in Anaconda v. 2–2.4.0 [34] was used for pre-processing data.

## Results

### Species identification

The current study was based on 264 isolates identified as *S. aureus* according to the reference method using MALDI-TOF MS analysis (Fig. 1). Using the 1928 platform, nine FASTQ files did not pass the internal quality control levels, since sequencing depth/coverage had to be higher than 30 × to perform the analysis (1928 Diagnostics, Sweden). Allowing depth/coverage of 11-29X for the species identification of these nine isolates, the 1928 platform showed 99.2% (262/264, 95% CI 97.1–99.9) agreement with the reference method. For the two discrepant results the 1928 platform predicted one of the isolates as *Staphylococcus epidermidis* (SA 310) whereas the second isolate (SA 1413) was predicted as non-staphylococcus spp. The INH also showed 99.2% (262/264, 95% CI 97.1–99.9) agreement to the reference method. Among the two discrepant results, the first isolate (SA 310) was also predicted as *S. epidermidis,* whereas the second isolate (SA 1413) was predicted as *Staphylococcus argenteus.* The two samples identified as *S. epidermidis* and *S. argenteus* were excluded from the rest of the study, since this study focused on *S. aureus.* Likewise, the FASTQ files that did not pass the internal quality control levels in the 1928 platform (1928 Diagnostics, Sweden) were excluded from further analysis. The upcoming benchmarking of virulence gene characterization, sequence type and antibiotic susceptibility included the remaining 255 *S. aureus* isolates.

### Antibiotic susceptibility test

In all, the EUCAST testing for the clinical *S. aureus* isolates generated 1006 phenotypic AST results (Table 4). The AST showed 2.5% (25/1006) isolates to be phenotypically resistant and the highest percentage of resistance was noticed for ciprofloxacin and fusidic acid (Table 4). Three out of 244 clinical isolates were phenotypically resistant to isoxazolyl penicillin (Table 4), being reported as cefoxitin resistant, which were further confirmed by in-house PCR as *mecA* gene positive and reported as methicillin resistant *S. aureus* (MRSA).

**Table 4:** Phenotypic antibiotic susceptibility test of *S. aureus* isolates

Antibiotic ^a^ (n) ^b^	Susceptible (n [%])	Resistant (n [%])
Ciprofloxacin (70)	66 [94.3]	4 [5.7]
Clindamycin (212)	209 [98.6]	3 [1.4]
Erythromycin (215)	212 [98.6]	3 [1.4]
Isoxazolyl penicillin (244)	241 [98.8]	3 [1.2]^c^
Rifampicin (5)	5 [100.0]	0 [0]
Trimethoprim (26)	25 [96.2]	1 [3.8]
Tetracycline (1)	0 [0]	1 [100.0]
Vancomycin (27)	27 [100.0]	0 [0]
Fusidic acid (206)	196 [95.1]	10 [4.9]
Total amount of cases: 1006	981 [97.5]	25 [2.5]

^a^Antibiotics reported are dependent on the set of antibiotics included for different sampling at the clinical lab (Additional file 1) and also the antibiotics included in the 1928 platform. ^b^Number of isolates tested with specific antibiotic disc during the EUCAST test. ^c^Followed by PCR detection of *mecA*, confirmation of MRSA

In order to study the capacity of the bioinformatic workflows to translate WGS into clinical practice within the area of AST, the degree of agreement between the predicted genotypic AST to the phenotypic AST result was studied (Table 5). There was an overall high agreement 98.0% (989/1006, 95% CI 97.3–99.0). Among the phenotypic AST results reported as susceptible 99.8% (979/981, 95% CI 99.2–99.9) of the results were concordant, while the phenotypic AST results reported as resistant were less concordant 60.0% (15/25, 95% CI 40.7–76.5) (Table 5). The degree of agreement between the predicted genotypic AST to the phenotypic AST result from 1928 and INH using ResFinder (Fig. 2) showed 99.0% (996/1006, 95% CI 98.1–99.5) and 98.4% (990/1006, 95%CI 97.4–99.0) agreement with the reference method. Lastly, comparison in-between the predicted genetic AST results showed a high overall agreement of 99.2% (998/1006, 95% CI 98.4–99.6). When looking closer into the nine different antibiotics selected for this study, there were 100% agreement across the phenotypic AST and the predicted genotypic AST for the following antibiotics reported as susceptible (S) or resistant (R): clindamycin S, erythromycin S, trimethoprim S and R, tetracycline S and R, rifampicin S and R, vancomycin S and R and isoxazolyl penicillin R (Table 5). In total, there were 26 discrepancies across the bioinformatic workflows including 23 VME and three ME, where fusidic acid showed the greatest discordance with 11 VME followed by clindamycin and ciprofloxacin showing six and four VME respectively, as well as one ME for ciprofloxacin (Table 5). Furthermore, discordance across the bioinformatic workflows resulting in VME could also be observed for erythromycin (n = 2) and ME for isoxazolyl penicillin (n = 2) (Table 5). Looking closer into the 26 discrepancies from the bioinformatic workflows (Table 6) one can notice that the output i.e., identified genetic resistance markers or genes from the tools included in the 1928 and INH workflows sometimes differs.

**Table 5:** Predicted genotypic AST results from the 1928 platform and the in-house pipeline compared to phenotypic AST

Antibiotic (n)^a^	Phenotypic AST (n)	Predicted genotypic AST from 1928 (n) and INH (n)				Discordant across methods (n [%])	Very major errors (n)		Major errors (n)	
		RR	SS	RS	SR		1928	INH	1928	INH
Ciprofloxacin (70)	R (4)	0	0	4	0	5 (7.1)	0	4	1	0
	S (66)	0	**65**	1	0					
Clindamycin (212)	R (3)	0	3	0	0	3 (1.4)	3	3	0	0
	S (209)	0	**209**	0	0					
Erythromycin (215)	R (3)	**2**	1	0	0	1 (0.47)	1	1	0	0
	S (212)	0	**212**	0	0					
Rifampicin (5)	R (0)	0	0	0	0	0 (0)	0	0	0	0
	S (5)	0	**5**	0	0					
Isoxazolyl penicillin (244)	R (3)	**3**	0	0	0	1 (0.41)	0	0	1	1
	S (241)	1	**240**	0	0					
Trimethoprim (26)	R (1)	**1**	0	0	0	0 (0)	0	0	0	0
	S (25)	0	**25**	0	0					
Tetracycline (1)	R (1)	**1**	0	0	0	0 (0)	0	0	0	0
	S (0)	0	0	0	0					
Vancomycin (27)	R (0)	0	0	0	0	0 (0)	0	0	0	0
	S (27)	0	**27**	0	0					
Fusidic acid (206)	R (10)	**3**	4	3	0	7 (3.4)	4	7	0	0
	S (196)	0	**196**	0	0					
Total n^b^ = 1006	S (981) R (25)	**10/**11	**979**/987	8	0	n^c^ = 17 (1.7)	n^d^ = 8	n^e^ = 15	n^f^ = 2	n^g^ = 1
Error rate %							0.8	1.5	0.2	0.1

The bioinformatic results are presented as 1928 platform/in-house pipeline (INH), where R/R is resistant/resistant, S/S is susceptible/susceptible, R/S is resistant/ susceptible, and S/R is susceptible/resistant

^a^Number of isolates tested with a specific antibiotic disc by disc diffusion. ^b^Total number of cases tested by disc diffusion. ^c^Number of discordant result implying both of the bioinformatic workflows. ^d^total very major error for 1928. ^e^Total very major error for INH. ^f^Total major error for 1928. ^g^Total major error for 1928, R-resistant; phenotypic AST R is retrieved from the EUCAST assay, genotypic AST R was conferred by the presence of resistance markers or genes, S-susceptible; phenotypic AST S was retrieved from the EUCAST assay, genotypic S was conferred by the absence of resistance markers and genes. INH-in-house pipeline using Resfinder. Very major error: resistant phenotype predicted as a susceptible genotype. Major error: susceptible phenotype predicted as resistant genotype. **Bold** 100% agreement with both bioinformatic tools

**Table 6:** Output from 1928 and INH where genotypic prediction of antibiotic susceptibility test showed VME (bold) or ME (underlined)

	**Disc diffusion** Phenotypic AST	**Bioinformatic workflows** Genotypic predicted AST	
Isolate	Antibiotic (R or *S*)	R or S Resistance markers identified by 1928	R or S Genes conferring antimicrobial resistance identified by INH
SA 907	Ciprofloxacin (R)	R *grlA* (S80F + I45M)^a^ *grlB* (E422D)	**S** **No genes**
SA 1525	Ciprofloxacin(R)	R *grlA* (S80F)	**S** **No genes**
SA 1852	Ciprofloxacin(R)	R *grlA* (S80F)	**S** **No genes**
SA 1153	Ciprofloxacin(R)	R *grlA* (S80F) *gyrA* (S84L)	**S** **No gene**
SA 1046	*Ciprofloxacin(S)*	R *grlA* (S80F + I45M) *grlB* (E422D)	S No genes
SA 61	Clindamycin(R)	**S** **No resistance markers**	**S** **No genes**
SA 63	Clindamycin(R)	**S** **No resistance markers**	**S** **No genes**
SA 804	Clindamycin(R)	**S** **No resistance markers**	**S** **No genes**
SA 804	Erythromycin(R)	**S** **No resistance markers**	**S** **No genes**
SA 535	Fusidic acid (R)	R *fusC*	**S** **No genes**
SA 846	Fusidic acid (R)	R *fusC*	**S** **No genes**
SA 1162	Fusidic acid (R)	R *fusC*	**S** **No genes**
SA 1333	Fusidic acid (R)	**S** **No resistance markers**	**S** **No genes**
SA 1349	Fusidic acid (R)	**S** **No resistance markers**	**S** **No genes**
SA 1640	Fusidic acid (R)	**S** **No resistance markers**	**S** **No genes**
SA 1370	Fusidic acid (R)	**S** **No resistance markers**	**S** **No genes**
SA 1197	*Isoxazolyl penicillin (S)*	R *mecC*	R *mecA*

^a^ () within brackets detected mutations are indicated. VME: Very major error: resistant phenotype predicted as a susceptible genotype. ME: Major error: susceptible phenotype predicted as resistant genotype. R-resistant; phenotypic AST R is retrieved from the EUCAST assay, genotypic AST R was conferred by the presence of resistance markers or genes, S-susceptible; phenotypic AST S was retrieved from the EUCAST assay, genotypic S was conferred by the absence of resistance markers and genes. INH-in-house pipeline using Resfinder

### Virulence genes and sequence type

We also compared the genetic predictions from the 1928 platform to those obtained by the INH concerning ST and presence (P) or absence (A) of selected virulence genes. In all, twenty-five genes were included in the analysis (Table 1). 1928 and the INH using VirulenceFinder (Fig. 2) showed overall 99.4% (1267/1275, 95% CI 98.7–99.7) agreement in genetic prediction of the chosen genes among the *S. aureus* (Table 7). The 1928 platform predicted the presence of another eight virulence genes than the INH using VirulenceFinder did, i.e., *etA* (n = 2), *etB* (n = 2) and *tsst1* (n = 4) (Table 7).

**Table 7:** Predicted presence (P) or absence (A) of certain virulence genes among 255 *S. aureus* isolates

Number of isolates and corresponding FASTQ files	Virulence gene of interest	Predicted genotype by 1928 platform and INH				Discordant across methods (n [%])
		AA^a^	PP^b^	AP	PA	
255	*etA*	251	2	0	2	2 [0.8]
255	*etB*	253	0	0	2	2 [0.8]
255	*lukF-PVL*	248	7	0	0	0 [0]
255	*lukS-PVL*	248	7	0	0	0 [0]
255	*tsst1*	215	36	0	4	4 [1.6]
Total (% of 1275)		1215 (95.3)	52 (4.1)	0 (0.0)	8 (6.3)	8 (6.3)

^a^A absence, ^b^P presence

In total, for 236 of the 250 isolates the results of MLST were consistent for the INH using MLST 2.0 (Fig. 2) and 1928 (Table 8). For 19 of the 255 isolates, the ST could not be definitely determined with either the INH or 1928. For three isolates, the ST was determined with 1928 but not with MLST 2.0 (SA117: ST50, SA656: ST5, and SA1723: ST39), while for two isolates, the ST was determined with MLST 2.0 but not with 1928 (SA331 and SA332, both ST6674). However, it can be noted that both workflows identified the same allelic profile for these two isolates even though 1928 did not yield any ST. Since typing of the SCCmec element is of importance to follow a possible MRSA outbreak, the 1928 platform also delivers a result about what kind of SCCmec element the *S. aureus* isolate is encoding. The 1928 platform predicted 17 isolates to encode for a SCCmec type, whereof four known and thirteen unknown types (Table 9). Among the four known SCCmec types, three of these were indeed predicted for the three isolates being phenotypic identified as MRSA in the clinical lab (SA 606, SA 1857 and SA 1153), but the clinical isolate, SA 1197, predicted to carry SCCmec XI was not. The 1928 platform genetically predicted the presence of *mecC*, while INH applying ResFinder predicted *mecA*, in the clinical isolate, SA 1197.

**Table 8:** Comparisons of STs as identified by INH (MLST-CGE) and 1928

ST	No. of isolates determined by			Discordant across methods [*n* (%)]
	Both INH and 1928	INH only	1928 only	
1	5	0	0	0 (0)
5	12	0	1	1 (7.7)
7	2	0	0	0 (0)
8	12	0	0	0 (0)
12	6	0	0	0 (0)
15	30	0	0	0 (0)
20	2	0	0	0 (0)
22	8	0	0	0 (0)
25	3	0	0	0 (0)
26	3	0	0	0 (0)
30	39	0	0	0 (0)
34	2	0	0	0 (0)
39	1	0	1	1 (50.0)
45	51	0	0	0 (0)
46	4	0	0	0 (0)
50	8	0	1	1 (11.1)
97	3	0	0	0 (0)
146	3	0	0	0 (0)
291	2	0	0	0 (0)
375	5	0	0	0 (0)
425	3	0	0	0 (0)
1181	2	0	0	0 (0)
1633	2	0	0	0 (0)
1693	3	0	0	0 (0)
6674	0	2	0^a^	2 (100.0)
Other STs^b^	20	0	0	0 (0)
Total (% of 236)^c^	231 (97.9)	2 (0.8)	3 (1.3)	5 (2.1)

CGE: Center for Genomic Epidemiology, INH: in-house pipeline, MLST: multi-locus sequencing typing, and ST: sequence type

^a^1928 identified same alleles as INH but assigned no ST

^b^Other STs include one isolate each of ST6, ST27, ST59, ST101, ST109, ST121, ST123, ST130, ST182, ST188, ST398, ST942, ST1021, ST1035, ST1150, ST1218, ST1675, ST2975, ST4554, and ST6363

^c^For 19 isolates, the ST profile could not be determined either by INH (MLST-CGE) or 1928. These isolates are not included in the table

**Table 9:** Extended genotypic prediction of the SCCmec type by 1928 platform among the 255 isolates

*S. aureus *isolate	ST		Sccmec type	IS1272	ccrA1	ccrA2	ccrA3	ccrA4	ccrB1	ccrB2	ccrB3	ccrB4	ccrB6	ccrC	mecA	mecC
	INH	1928														
SA 606	375	375	IV	✓		✓				✓					✓	
SA 1857	30	30	IV	✓		✓				✓					✓	
SA 1153	5	5	V/VII											✓	✓	
SA 1197	130	130	XI		✓						✓					✓
SA 1637	ND	ND	Unknown	✓												
SA 112	398	398	Unknown	✓												
SA 1725	22	22	Unknown	✓												
SA 1752	5	5	Unknown					✓				✓				
SA 1828	ND	ND	Unknown			✓				✓						
SA 1896	34	34	Unknown		✓						✓					
SA 998	5	5	Unknown	✓												
SA 215	5	5	Unknown	✓												
SA 261	1	1	Unknown	✓												
SA 365	182	182	Unknown			✓				✓						
SA 1162	1	1	Unknown		✓				✓							
SA 535	1	1	Unknown		✓				✓							
SA 846	1	1	Unknown		✓				✓							

ND: ST profile could not be determined

## Discussion

In this study, the 1928 platform was benchmarked to reference methods in the clinical laboratory as well as to an in-house developed bioinformatic pipeline (Fig. 2). Among all bacterial isolates collected during the prospective observational study of community-onset severe sepsis and septic shock [8], *S. aureus* was one of the most common etiological agents among the patients suspected of having sepsis.

### Species identification

Currently the INH includes the tool JSpeciesWS (Fig. 2), calculating the pairwise ANI against reference genomes [28] for prediction of species identification. Recently, another study [19], using similar preprocessing tools for FASTQ PE files retrieved from Illumina sequencing, also showed good achievement in species identification using the JSpeciesWS tool. Nevertheless, during the development of the INH, different tools for prediction of species identification were assessed i.e., 16S rRNA based species identification of *S. aureus* using CGE SpeciesFinder [35], kmer-based species identification with kmer size 16 and prefix “ATG” with CGE KmerFinder [35–37]. Also, species discrimination application of dDDH based on the Type (Strain) Genome Server, TYGS [38] was assessed. CGE Kmerfinder and TYGS showed high agreement with the reference method 99.2% (262/264), while SpeciesFinder predicted only 76.5% (202/264) as *S. aureus* (Additional file 2). Similar challenges have been reported earlier [35]. The 1928 platform analysis for species identification, applying assembly free kmer-based method, showed 99.2% (262/264) agreement to the reference method. It should however be emphasized that depth/coverage of 11-29X were allowed for the species identification of nine isolates. Including only the FASTQ files passing 1928 internal quality threshold, the 1928 platform would have shown 100% (255/255) agreement to the reference method. Among the jointly predicted discrepant results (2/264), one isolate (SA 310) was predicted to be *S. epidermidis* by both JSpeciesWS and 1928, while the second isolate (SA 1413) was predicted as non-staphylococcus spp. by 1928, whereas JSpeciesWS predicted *S. argenteus.* In 2015, two novel species of the genus *Staphylococcus* were identified by WGS using the Illumina HiSeq platform, where one was proposed as *S. argenteus* [39]. The general clinical impact of *S. argenteus* is difficult to assess because of the limited number of studies and datasets, and divergent observations exist, but recent studies suggest that the frequency of healthcare-associated infections, morbidity and mortality are comparable to those of *S. aureus* [40, 41]. In addition, there have been multiple reports of bloodstream infections among which *S. argenteus* methicillin resistant isolates have been isolated [40, 42–44], altogether illustrating the importance of *S. argenteus* identification. To date, classical routine diagnostics do not distinguish this species from *S. aureus* [45]. Though, since April 2018 the clinical microbiology laboratory Unilabs, Skövde, is using the updated Bruker microorganism database MBT Compass Library DB-7854 (Bruker Daltonics, Germany) including identification of *S. argenteus.* Shortly after all WGS data collected in our study had been analyzed, the very first report of *S. argenteus* in Sweden was published and the 1928 platform was updated accordingly [16].

### Antibiotic susceptibility test

Combined predicted genotypic antibiotic susceptibility from both of the bioinformatic workflows showed 98.0% (989/1006, 95% CI 97.3–99.0) agreement to phenotypic AST (Table 5), which has also been shown in other studies for *S. aureus* [9, 13, 46, 47]. The phenotypic AST results reported as resistant were less concordant than the phenotypic AST results reported as susceptible (Table 5). Similar results have been reported, when studying the accuracy of three different bioinformatic systems Genefinder, Mykrobe and Typewriter in genetic prediction of AST from *S. aureus* WGS data [9].

These bioinformatic systems showed challenges in concordant genetic predicted AST with phenotypic AST for the antibiotics, ciprofloxacin and fusidic acid, which was also the case in our study (Table 5). Among the 26 discrepancies across the bioinformatic workflows fusidic acid showed the greatest discordance with 11 VME followed by clindamycin and ciprofloxacin showing six and four VME respectively, as well as one ME for ciprofloxacin (Table 5). The discrepancies reported for the fusidic acid comes from the 1928 platform predicting the presence of *fusC* in three isolates (SA 535, SA 846 and SA 1162), while the INH did not predict the presence of any genes, resulting in three VME for the INH. Also, four isolates reported to be phenotypic resistant to fusidic acid (SA 1333, SA 1349, SA 1640 and SA 1370) were genotypically predicted susceptible since no gene or resistance markers could be predicted by the bioinformatic workflows (Table 6), resulting in four VME for each bioinformatic workflow. The resistance mechanism by fusidic acid, inhibiting protein synthesis, has been shown to have multiple genetic causes, some of which have only recently been discovered [48], illustrating the need for recognition of novel variants in the systems database for in silico prediction of resistance and susceptibility. For ciprofloxacin four isolates reported to be phenotypic resistant (SA 907, SA 1525, SA 1852 and SA 1153) the INH did not predict the presence of any gene, while the 1928 platform predicted the isolates to be resistant by the presence of several different genotypic resistance markers (Table 6), resulting in four VME for INH. Also, isolate SA 1046, reported to be phenotypic susceptible to ciprofloxacin was predicted by 1928 to be resistant by the presence of different genotypic resistance markers, while the INH could not predict any genes (Table 6), resulting in one ME for the 1928 platform. Even though only 1.0% of cases were reported in this study as phenotypically resistant for clindamycin, the bioinformatic workflows predicted all of them to be genotypically susceptible, resulting in six VME (Table 5). Bioinformatic tools showing concordant predictions for clindamycin but disagreed with phenotypic AST for *S. aureus* have also been reported in other studies [9, 49]. Likewise, previous studies of clindamycin resistance have reported positive *ermC* PCR results from nondetectable resistance phenotypes, suggesting that plasmids conferring resistance to these antibiotics may be lost in subculture, and therefore not present in the WGS data [46, 50]. ME reported for clindamycin may be inducible clindamycin resistant not detected by current phenotypic methods, but present in the WGS data. Since there has recently been evidence for increased worldwide inducible clindamycin resistance [51, 52], the bioinformatic workflows should consider this antibiotic group and continue the development of algorithm taking these identified challenges into account. For erythromycin, three isolates (SA 61, SA 63 and SA 804) were phenotypic resistant and both bioinformatic workflows predicted the presence of *ermC* in two isolates (SA 61 and SA 63), but the output from the bioinformatic workflows did not tell if it was plasmid mediated. The third isolate, SA 804, no resistance markers or genes was predicted by the bioinformatic workflows, resulting in two VME (Table 6). Since the antibiotic group fusidic acid had most VME (n = 11), the highest VME rate was also identified for fusidic acid, where 1928 reported 1.9% (4/206) and INH 3.4% (7/206), followed by clindamycin, where both bioinformatic workflows reported a VME rate of 1.4% (3/212), and for ciprofloxacin the 1928 platform showed a VME rate of 1.4% (1/70) and the INH 5.7% (4/70) (Table 5). Other studies using bioinformatic workflows such as blastn and tblast [13] and Next Gen Diagnostic [12] showed similar VME rates of 1.4% and 1.2% for ciprofloxacin respectively, while higher VME rates for clindamycin has been reported when using the 1928 platform i.e., 8.8% and 5.9% when using Next Gen Diagnostics [49]. Discordant AST genotypic predictions could be due to different algorithms being employed by the bioinformatic tools, demonstrating the need of international agreement on quality control. Only data sets passing agreed quality control metrics should be used in antimicrobial susceptibility predictions as resistance genes or mutations otherwise might be missed in sequences of poor quality [53]. In this study 100% (264/264) and 96.6% (255/264) FASTQ PE-files passed internal quality control metrics used by the INH and the 1928, respectively. The discordant AST genetic predictions for ciprofloxacin and fusidic acid was probably due to differences in the resistance database for the two bioinformatic workflows (Table 6). Nevertheless, the other discordant results cannot be deducted if the discordant AST genetic predictions were attributed to differences in the resistance database or the combination of assembly + BLAST within ResFinder 2.0 versus the assembly-free kmer-based method of 1928. A recent systematic review, using the CARD database 3.0.3 and Resfinder 4.0 on data retrieved from only Gram-negative bacteria, suggested the complexity of connecting genotype to phenotype with factors not yet considered in the resistance databases, for example gene regulation etc. [15]. Individually, both 1928 and INH using Resfinder demonstrated high agreement with the phenotypic AST (Table 5). However, among the discordant results for each bioinformatic workflow, the 1928 platform showed lower VME rate than the INH using Resfinder, 0.8% (8/1006) versus 1.5% (15/1006), while the ME rate was slightly higher for the 1928 platform compared to the INH 0.2% (2/1006) versus 0.1% (1/1006). This is of importance, since VME, false negatives, might result in use of an ineffective therapeutic agent for treatment, leading to treatment failure, while a ME might limit therapeutic options and complicate treatment [14, 15].

### Virulence genes and sequence type

Identification of *S. aureus* virulence genes can give the clinician insight into an infection’s pathogenesis and supporting the choice of therapy [54, 55]. The list of virulence genes that 1928 detects (Table 1) has been formed by requests from the platform's users. The exfoliative toxins, encoded by *etA* and *etB* are the cause for staphylococcal scalded skin syndrome [56]. The *tsst1* gene, encoding the toxic shock syndrome toxin-1, may cause staphylococcal toxic shock syndrome [57]. The Panton-Valentine leucocidin (PVL) exotoxin, encoded by the *lukF-PVL* and *lukS-PVL* genes, is associated with *S. aureus* infections and is linked to infection severity and outcome in invasive disease [58]. During the time the study took place the clinical lab did not perform any reference method for identification of these genes or expected phenotype, such as agglutination or ELISA assays for detection of toxic shock syndrome toxin-1 [59, 60]. Therefore, no benchmarking with results from the clinical lab can be addressed. The bioinformatic workflows showed overall high agreement in the genetic prediction of the virulence traits (Table 7). The *S. aureus* isolates collected during this study showed highest genetic predicted frequencies of the *tsst1* and *lukF-PVL, lukS-PVL* genes (Table 7)*.* The 1928 platform predicted the *tsst1* and *lukF-PVL, lukS-PVL* to be present among 15.7% (40/255) and 2.7% (7/255) of the *S. aureus* isolates, while INH using VirulenceFinder predicted the *tsst1* and *lukF-PVL, lukS-PVL* to be present among 14.1% (36/255) and 2.7% (7/255) of the *S. aureus* isolates. Another epidemiological marker is typing of the SCCmec element, aiding in understanding the evolution of MRSA and to follow a possible MRSA outbreak. The 1928 platform deliver a result about what kind of SCCmec element the *S. aureus* isolate is encoding, by including genetic prediction of thirteen different genes from the SCCmec casettes (Table 1). The platform predicted four known SCCmec types whereof the *S. aureus* isolate (SA 1197) predicted to belong to SCCmec type XI, have a cassette containing the recently identified *mecC* gene [61, 62] (Table 9). This isolate was identified as phenotypic susceptible to isoxazolyl penicillin, explaining the discordant result between the predicted genotypic AST results by both bioinformatic workflows (1/244, 0.4%) compared to the phenotypic AST (Table 5). In Sweden, the first MRSA with *mecC* was isolated in 2003 from a hedgehog but was not described as *mecC* until 2012 [63]. Since 2012 the Swedish Communicable Diseases Act has been including *S. aureus* with *mecC* as a mandatory notifiable disease and handled in the same way as *S. aureus* with *mecA* regarding follow-up and contact tracing among household and healthcare contacts. Therefore, the current recommended routine diagnostics is to include a PCR assay for simultaneous detection of *mecA* and *mecC* [64] if a *S. aureus* isolate is resistant or intermediate resistant to the β-lactam cefoxitin. Nowadays, the prevalence of human *mecC*-MRSA infections is very low. However, *mecC*-MRSA isolate transmission between different hosts indicates the great capacity of these isolates for spreading and still the possible impact that these isolates can have in clinical settings remains unknown [65]. In the SCCmec types annotated as unknown by 1928, the *ccrA* and *ccrB* genes were found, but *mecA* and *mecC* were absent. The absence of *mecA*/*mecC* agreed with the phenotypic AST which showed susceptibility to isoxazolyl penicillin of these isolates. It can be likely that these isolates harbor SCCmec remnants where they have lost the *mecA* and *mecC* genes, as has been observed in an earlier study [66]. Another method being used to investigate the relationship between pathogens, but more on a global level, is by MLST and determination of ST. The 1928 platform showed overall 97.9% (231/236, 95% CI 95.0–99.2%) agreement with the INH using MLST 2.0 in predicting STs of the *S. aureus* isolates (Table 7). However, it should also be noted that for 19 isolates, the ST could not be definitely determined with the INH using MLST 2.0 or 1928. Comparison of classical MLST software for NGS data, have shown that not all MLST applications function as expected. MLST 2.0 was one of the tools used [67]. Problems with some software included: poorly updated databases, computationally inefficient methods, false-positive results, inability to call alleles at low coverage and variable performance in the presence of mixed samples [67]. Therefore, there is scope for improvement.

### Time and user-friendliness

For WGS to be adopted in infection control and public health, it is required to be fast and generate robust results regarding the genomic context. Indeed, both of the bioinformatic workflows showed reliable results by demonstrating high agreement with the results retrieved in clinical routine, but there were differences in processing times between the bioinformatic workflows. The INH is code-level workflow, requiring formal bioinformatic support for operation and included steps of quality control followed by downstream analysis of the sequencing data. Estimated computational time required for analysis of one bacterial isolate, including two FASTQ PE files as input to the INH (Fig. 2) was 5–6 h using the Intel(R) Core (TM) i5-6300U CPU @ 2.40 GHz 2.40 GHz, RAM 16 GB, 64-bit PC. In more detail, the preprocessing of the FASTQ PE files took about 15 min, assembly and scaffolding about 2–3 h and finally annotation about 3 h, but time increased with queue size for the CGE webserver. The user needed to make a manual summary of the retrieved output. Using the same computer power, the computational time for the 1928 platform was 15–30 min. The raw sequencing data were directly uploaded and processed by the 1928 platform and the user received a summary of the retrieved output. Nevertheless, the FASTQ files that did not pass the internal quality control took about 24 h before the failed result was reported. Although being very user-friendly, a limitation with 1928 is that the user is restricted to the analyses included in the platform as opposed to the INH pipeline which can be extended with additional analyses available on the CGE and JSpeciesWS. There are also possibilities to extend the genotypic AST tools for the INH, since there are several freely accessible bioinformatics resources for detection of antimicrobial resistance determinants in DNA or amino acid sequence data, so far, e.g. ARG-ANNOT, CARD, SRST2, MEGARes, Genefinder, ARIBA, KmerResistance and AMRFinder [68].

## Conclusions

Altogether, the benchmarking revealed that both bioinformatic workflows deliver results with high accuracy aiding diagnostics of severe infections caused by *S. aureus,* while the ST of *S. aureus* show scope for improvement. Our study is validating the performance of the 1928 platform in clinical routine for in silico species identification, antibiotic susceptibility testing and virulence profiling. The 1928 platform is also suitable for use in a clinical laboratory, since it is more user-friendly and deliver results timely. Still genotypic predictions cannot yet replace the phenotypic tests as in silico AST prediction for other organisms has been proved more challenging, especially for Gram-negative bacteria [19, 69] where the present understanding of genetic basis of resistance is less comprehensive. Additionally, the standardization of WGS workflows is a central requirement when entering the clinical diagnostics.

## Supplementary Information

**Additional file 1. **Specification of antibiotics used in the phenotypic antibiotic susceptibility testing for* S. aureus *isolates as part of the routine practice in the clinical laboratory. Phenotypic antibiotic susceptibility testing results reported in this study are limited from the set of antibiotics included in the 1928 platform.

**Additional file 2. **Genetically predicted species identification using bioinformatic tools for the 264 isolates identified as* S. aureus *by MALDI-TOF MS.

## Data Availability

The datasets generated and/or analysed during the current study are available in the online NCBI repository, https://www.ncbi.nlm.nih.gov/, BioProject PRJNA606666, http://www.ncbi.nlm.nih.gov/bioproject/606666

## Abbreviations

AST: Antibiotic susceptibility testing
ST: Sequence types
NGS: Next-generation sequencing
WGS: Whole-genome sequencing
INH: In-house developed bioinformatic pipeline
MLST: Multi-locus sequence typing
VME: Very major error
ME: Major error
MRSA: Methicillin resistant *S. aureus*
EUCAST: European Committee on Antimicrobial Susceptibility Testing
S: Susceptible
R: Resistant
P: Presence
A: Absence
PVL: Panton-Valentine leucocidin
CGE: Center for Genomic Epidemiology
