# Supplementary material for: Benchmarking of two bioinformatic workflows for the analysis of whole-genome sequenced Staphylococcus aureus collected from patients with suspected sepsis
Source: BMC Infect Dis. 2023 Jan 20;23:39. doi: 10.1186/s12879-022-07977-0 (PMC9863170; doi:10.1186/s12879-022-07977-0)
Supplement: Supplementary file 1 — Additional file 1. Specification of antibiotics used in the phenotypic antibiotic susceptibility testing for S. aureus isolates as part of the routine practice in the clinical laboratory. Phenotypic antibiotic susceptibility testing results reported in this study are limited from the set of antibiotics included in the 1928 platform. [file 12879_2022_7977_MOESM1_ESM.docx]

Additional file 1

Specification of antibiotics used in the phenotypic antibiotic susceptibility testing for *S. aureus* isolates as part of the routine practice in the clinical laboratory. Phenotypic antibiotic susceptibility testing results reported in this study are limited from the set of antibiotics included in the 1928 platform

| **Antibiotic**^a^ | **Sample type** | | | | | | |
| --- | --- | --- | --- | --- | --- | --- | --- |
|  | Routine swab | Blood | Lower respiratory | Upper respiratory | Urine | Punctuate  Liquid | Peritoneal liquid |
| **Quinolone** |  |  |  |  |  |  |  |
| Ciprofloxacin | ● | ● | ● | ● | ● | ● | ● |
| **Lincosamide** |  |  |  |  |  |  |  |
| Clindamycin | ● | ● | ● | ● | ● | ● | ● |
| **Macrolide** |  |  |  |  |  |  |  |
| Erythromycin | ● | ● | ● | ● | ● | ● |  |
| **β-lactam** |  |  |  |  |  |  |  |
| Isoxazolyl penicillin | ● | ● | ● | ● | ● | ● |  |
| **Rifampicin** |  |  |  |  |  |  |  |
| Rifampicin | ● | ● |  | ● |  |  |  |
| **Tetracycline** |  |  |  |  |  |  |  |
| Tetracycline | ● | ● | ● | ● | ● | ● | ● |
| **Trimethoprim** |  |  |  |  |  |  |  |
| Trimethoprim | ● |  |  | ● | ● |  |  |
| **Glycopeptide** |  |  |  |  |  |  |  |
| Vancomycin | ● | ● | ● | ● | ● | ● | ● |
| **Fusidic acid** |  |  |  |  |  |  |  |
| Fusidic acid |  |  |  | ● |  |  |  |

^a^Phenotypic AST results reported in this study are limited from the set of antibiotics included in the 1928 platform. ^b^Intermediate and resistant AST result for isoxazolyl penicillin was followed by detection of *mecA* by PCR to confirm the isolate as a methicillin resistant *S. aureus* (MRSA).
