# Supplementary material for: Benchmarking of two bioinformatic workflows for the analysis of whole-genome sequenced Staphylococcus aureus collected from patients with suspected sepsis
Source: BMC Infect Dis. 2023 Jan 20;23:39. doi: 10.1186/s12879-022-07977-0 (PMC9863170; doi:10.1186/s12879-022-07977-0)
Supplement: Supplementary file 2 — Additional file 2. Genetically predicted species identification using bioinformatic tools for the 264 isolates identified as S. aureus by MALDI-TOF MS. [file 12879_2022_7977_MOESM2_ESM.docx]

**Additional file 2**

Genetically predicted species identification using bioinformatic tools for the 264 isolates
identified as *S. aureus* by MALDI-TOF MS.

| **Bioinformatic tool** | ***S. aureus* n [%]** | **Other species  n [%]** | **No prediction**  **n [%]** |
| --- | --- | --- | --- |
| JSpeciesWS | 262 [99.2] | 2 [0.8]^a^ | 0 [0] |
| SpeciesFinder | 202 [76.5] | 1 [0.4]^b^ | 61 [23.1]^c^ |
| TYGS | 262 [99.2] | 2 [0.8]^a^ | 0 [0] |
| KmerFinder | 262 [99.2] | 2 [0.8]^a^ | 0 [0] |
| 1928 | 262 [99.2] | 2 [0.8]^d^ | 0 [0] |

^a^*S. epidermidis* and *S. argenteus,* ^b^*S*. *epidermidis,* ^c^Results lower than 98% ID match were excluded, ^d^*S. epidermidis* and unknown.
